# Early developmental stages of native populations of *Ciona intestinalis* under increased temperature are affected by local habitat history

**DOI:** 10.1242/jeb.233403

**Published:** 2021-03-05

**Authors:** Elizabeth A. Clutton, Gaston Alurralde, Tiago Repolho

**Affiliations:** 1 Institute of Marine Sciences, Faculty of Science and Health, University of Portsmouth, Eastney, Portsmouth PO4 9LY, UK; 2 Universidad Nacional de Córdoba, Facultad de Ciencias Exactas, Físicas y Naturales, Departamento Diversidad Biológica y Ecología, Ecología Marina, Av. Velez Sarsfield 299 (X5000JJC), Córdoba, Argentina; 3 Consejo Nacional de Investigaciones Científicas y Técnicas (CONICET), Instituto de Diversidad y Ecologıa Animal (IDEA), Av. Velez Sarsfield 299 (X5000JJC), Córdoba, Argentina; 4 MARE - Centro de Ciências do Mar e do Ambiente (MARE), Faculdade de Ciências, Universidade de Lisboa, 1749-016 Lisboa, Portugal

**Keywords:** Ascidians, Biological invasion, *Ciona intestinalis*, Early life stages, Marine heatwave, Ocean warming

## Abstract

Temperature modulates marine ectotherm physiology, influencing survival, abundance and species distribution. While native species could be susceptible to ocean warming, thermal tolerance might favour the spread of non-native species. Determining the success of invasive species in response to climate change is confounded by the cumulative, synergistic or antagonistic effects of environmental drivers, which vary at a geographical and temporal scale. Thus, an organism's acclimation or adaptive potential could play an important evolutionary role by enabling or conditioning species tolerance to stressful environmental conditions. We investigated developmental performance of early life stages of the ascidian *Ciona intestinalis* (derived from populations of anthropogenically impacted and control sites) to an extreme weather event (i.e. marine heatwave). Fertilization rate, embryo and larval development, settlement, metamorphosis success and juvenile heart rate were assessed as experimental endpoints. With the exception of fertilization and heart rates, temperature influenced all analysed endpoints. *C. intestinalis* derived from control sites were the most negatively affected by increased temperature conditions. By contrast, *C. intestinalis* from anthropogenically impacted sites showed a positive response to thermal stress, with a higher proportion of larvae development, settlement and metamorphosis success being observed under increased temperature conditions. No differences were observed for heart rates between sampled populations and experimental temperature conditions. Moreover, interaction between temperature and populations was statistically significant for embryo and larvae development, and metamorphosis. We hypothesize that selection resulting from anthropogenic forcing could shape stress resilience of species in their native range and subsequently confer advantageous traits underlying their invasive potential.

## Abstract (precis)

**Summary:** Anthropogenic stressors can shape early developmental stages of *Ciona intestinalis*, increasing their resilience to predicted future heatwave scenarios when compared with control populations from undisturbed locations.

## INTRODUCTION

Extreme weather events are becoming more frequent and prolonged over time, pushing Earth's climate beyond its natural variability (Meehl and Tebaldi, 2004; Herring et al., 2016; Scannell et al., 2016; Mann et al., 2017; Oliver et al., 2018). Within the extreme weather events category, marine heatwaves, defined as an elevation above mean sea surface temperature of 3–5°C for at least 3 days (Sorte et al., 2010), have been identified as major climate change stressors (Hobday et al., 2018; Smale et al., 2019; Holbrook et al., 2019). Over the past 126 years, shorter lived but more intense heatwaves have doubled in length (Della-Marta et al., 2007). Moreover, by the late 21st century, the persistence of long-lived heatwaves (i.e. lasting approximately 1.5 weeks) in central western Europe is estimated to be around 50% longer in comparison with the 1961–1990 timescale (Meehl and Tebaldi, 2004). Nearly 38% of the world's coastline has experienced an increase of anomalously high seawater temperature events (Lima and Wethey, 2012), with documented negative impacts on species survival and community/population structure (Schiel et al., 2004; Garrabou et al., 2009; Grilo et al., 2011; Wernberg et al., 2013; Pansch et al., 2018; Seuront et al., 2019; Saha et al., 2020). Marine heatwaves and the resulting ocean warming pose deleterious effects over species physiology (Diez et al., 2012; Rius et al., 2014; Stillman, 2019), besides affecting processes to which species growth and reproduction depend upon (Vilchis et al., 2005; Le Bohec et al., 2008; DeCarlo et al., 2017). Temperature is one of the most important abiotic factors influencing marine species survival, abundance and distribution, and on which species physiology is highly dependent (Pörtner and Knust, 2007; Somero, 2010; Aurélio et al., 2013; Repolho et al., 2014, 2017; Rosa et al., 2016; Kenworthy et al., 2018).

Physiological performance of a species reflects adaptation to a particular environment (Magozzi and Calosi, 2015), local adaptation or acclimation to environmental related pressures, and could play an important evolutionary role to confer tolerance to varying environmental conditions (Renborg et al., 2014). A high physiological tolerance to ambient conditions might enable a greater potential to survive, migrate and subsequently colonize new sites (Rius et al., 2010; Gröner et al., 2011; Rocha et al., 2017; Gewing et al., 2019; Kenworthy et al., 2018). This is particularly crucial at early life stages of development (Hamdoun and Epel, 2007) with regard to sessile species that are unable to escape from harsh conditions, beyond metamorphosis (Ng and Keough, 2003; Bellas et al., 2004; Gewing et al., 2019). In this sense, phenotypic plasticity may allow organisms to persist in the face of environmental change and give populations the time to adapt to climate change (Chevin et al., 2010). However, plasticity depends not only on the conditions experienced by organisms during their lifetime, but can also depend on the conditions experienced by previous generations (Marshall and Uller, 2007; Burgess and Marshall, 2011; Lange and Marshall, 2017). Transgenerational effects of the environmental conditions experienced by its parents will determine an offspring's performance, either positively (Marshall and Morgan, 2011) or negatively (Salinas and Munch, 2012). It is therefore probable that not all populations spread through different climatic regimes will respond in the same way to environmental pressure (Calosi et al., 2017; Peck, 2018). This may create spatial patterns of variable susceptibility of marine communities to future environmental conditions (Burgess and Marshall, 2011; Donelson et al., 2012; Miller et al., 2012). From this perspective, population-specific tolerance originating from harsh and fluctuating environments (Lenz et al., 2011), together with the interaction between environmental conditions and parental experience, could determine offspring fitness (Bernardo, 1996; Marshall and Uller, 2007; Lange and Marshall, 2017). Thus the reciprocal exchange between species-specific adaptations and site-specific environmental characteristics and the ability to withstand a broad and fluctuating range of environmental conditions would be key for some organisms to become successful invaders (Facon et al., 2006; Richards et al., 2006; Lenz et al., 2011; Marie et al., 2017; Rocha et al., 2017).

The combination of adaptive capacity and history of exposure may enable selection for greater plasticity in novel environments, with resulting tolerance towards different environmental stressors enhancing the invasive potential of certain species. Therefore, evolutionary histories of different populations of a given species may result in a differential invasive potential challenging our understanding of invasion processes (Bock et al., 2012; Gewing et al., 2019; Chen et al., 2018). Empirical evidence of how extreme weather events (i.e. heatwaves) can affect early life stages of development remains poorly understood (Diez et al., 2012). Thus, considering the consequences of marine heatwaves and the potential selection occurring as a result of exposure to anthropogenically impacted environments, one could hypothesize that populations of the same species, developed under different environmental conditions, may demonstrate varied resilience during early life stage development.

The biology and ecology – including population ecology – of the solitary ascidian *Ciona intestinalis* (Linnaeus 1767) is well described within Scandinavian coastal areas (Dybern, 1965; Svane and Havenhand, 1993). In this region, this species occurs in its putative native range, both in anthropogenic-impacted and undisturbed environments (Brunetti et al., 2015). As a native species within the west coast of Sweden, *C. intestinalis* is a suitable biological system to explore the impact of marine heatwaves on early life stage development of populations subjected to different habitat history. Although, connectivity among *C. intestinalis* populations can modulate the flux of adaptive genetic variation from numerous areas and habitats (Ghabooli et al., 2013), the ability of *C. intestinalis* to disperse is crucially dependent on larval competence and capability to settle (Rius et al., 2014). At the local scale, short-distance dispersal occurring by means of drifting *C. intestinalis* eggs, egg-strings and swimming larvae (Svane and Havenhand, 1993) limit the dispersion of this species, once the eggs and developing larvae are maintained in mucus strings (Petersen and Svane, 1995) and local oceanographic patterns constrain *C. intestinalis* populations connectivity (Johannesson et al., 2018). Early life stages of *C. intestinalis* are sensitive to heat stress, exhibiting a thermal response within a temperature range of 8–22°C (Dybern, 1965). During embryogenesis heat-inducible gene expression, like the heat shock protein Ci-HSPA1/6/7-like, is negligible under normal conditions, while it is highly expressed when cells are exposed to higher temperatures (from 23 to 28°C), which represents a protective mechanism for the organism under heat stress (Kawaguchi et al., 2015). How environmental background affects species resilience towards heat stress, should be further investigated, considering the notable recruitment ability of *C. intestinalis*, its adaptability towards environmental variation as well as its global success as an invasive species (Therriault and Herborg, 2008; Zhan et al., 2010; Rocha et al., 2017; Kenworthy et al., 2018). In the sister species *Ciona* *robusta*, a rapid microevolutionary process has been shown to be responsible for the harsh environmental adaptation and therefore contributes to invasion success in different aquatic ecosystems with largely varied environmental factors (Chen et al., 2020). Thus, understanding the tolerance of species to environmental conditions is the key to understanding their potential spread and the ability to escape from their native range and become invasive. Thus, determining the ecological effects of anthropogenic stressors and the potential for resistance or resilience enables more informed environmental management decisions.

Anthropogenic and environmental stressors often occur in combination, thus in modified coastal systems, organisms will experience both water contamination/pollution and environmental stress. Anthropogenically impacted environments can contain contaminants/pollutants such as metals and chemicals associated with antifouling paints, as reported for small harbours in west Sweden (Eklund et al., 2016; Gustavsson et al., 2017). Such chemical and heavy metal exposure is known to have detrimental impacts on *C. intestinalis* (Gallo et al., 2011; Gallo and Tosti, 2013; Gallo and Tosti, 2015) and its sister species *C.* *robusta* (Caputi et al., 2019). The impact of environmental contamination can lead to significant differences in species and community composition between marinas and that of undisturbed sites (Kenworthy et al., 2018). Whilst the present study does not set out to quantify environmental contaminants, the sample sites have been described as either anthropogenically impacted or undisturbed depending on the proximity to a marina.

The present study aimed to understand if the local habitat history of different *C. intestinalis* populations could shape stress resilience of their early life stages to an extreme temperature increase (i.e. simulating a marine heatwave). Heatwaves are expected to rise in frequency and intensity (Meehl and Tebaldi, 2004) and in some regions the increase can be much higher, in particular in the North Sea (Belkin, 2009). We selected five different site locations that we consider to be isolated populations, owing to limited larvae dispersal. The locations selected represent a broad range of environments, from relatively undisturbed sites located along the shores of the fjord, and also selected organisms from within three marinas. Developmental performance of *C. intestinalis*, derived from populations with different life histories (i.e. anthropogenically impacted versus control sites) was assessed under two temperature conditions, i.e. control (17°C, monthly average sea-surface temperature recorded for the studied area) and increased temperature conditions (22°C, marine heatwave scenario). Six response-related endpoints were analysed: (i) fertilization success; (ii) embryo development; (iii) larva development (i.e. competent larvae); (iv) settlement success; (v) metamorphosis success and (vi) juvenile heart rate.

## MATERIALS AND METHODS

### Sampling site

**Fig. 1.**
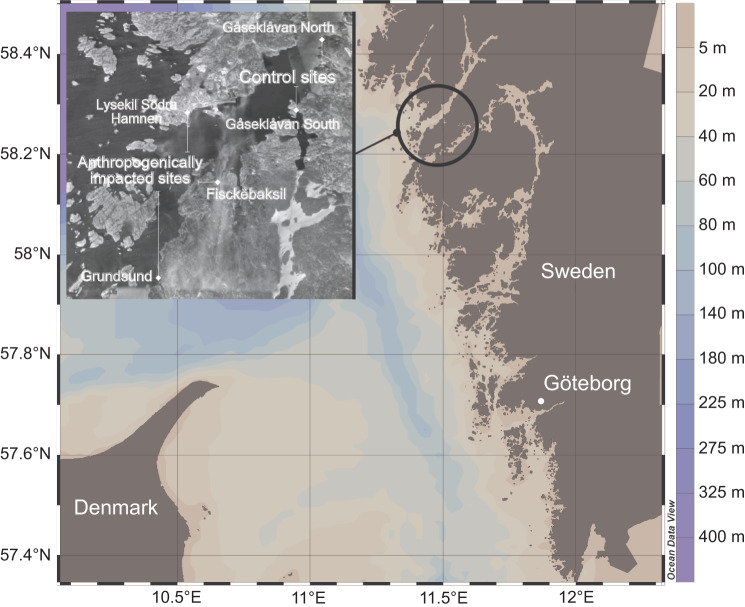
***Ciona intestinalis* sampling sites (inset map) (*n*=5) located in the Gullmarsfjörden (Sweden).** Control sites: Gåseklåvan North and Gåseklåvan South. Anthropogenically impacted sites: Fisckëbaksil, Grundsund and Lysekil Södra Hamnen. Map adapted from Ocean Data View (http://odv.awi.de).

*Ciona intestinalis* were collected by SCUBA diving at 3–10 m depth in the Swedish coastal shore, at five sites located in the Gullmarsfjörden (Fig. 1, 58°15′27.6″N, 11°26′13.2″E). The five geographically distinct sites included two control (Gåseklåvan North, 58°19′15.09″N, 11°32′01.10″E; and Gåseklåvan South, 58°18′19.09″N, 11°32′12.29″E) and three anthropogenically impacted locations, inhabiting marinas (Fisckëbaksil, 58°24′43.25″N, 11°46′13.26″E; Grundsund, 58°21′47″N, 11°41′67″E; and Lysekil Södra Hamnen 58°27′06.21″N, 11°43′64.70″E). Small boat harbors on the west coast of Sweden, have been shown to be heavily contaminated/polluted by copper, zinc, butyltins, polycyclic aromatic hydrocarbons (PAHs), and to a lesser extent by lead (Eklund et al., 2016). Marina sites near Fisckëbaksil were chosen as anthropogenically impacted areas since chemical-derived contamination/pollution associated with boat traffic and maintenance activities have been reported here in concentrations above their natural levels (Schiff et al., 2004; Gustavsson et al., 2017). Besides metals and PAHs, contaminants in the area include anionic surfactants, phthalate esters, chlorinated volatile organic compounds and petroleum residues (Gustavsson et al., 2017). Even though the boating pressure today might be high enough to produce toxic effects even in natural harbours in pristine areas, natural harbours are predicted to have fewer contaminants/pollutants and be less toxic than the small boat harbours (Eklund et al., 2016). It is therefore predicted that, *C. intestinalis* inhabiting anthropogenically impacted areas, are exposed to anthropogenically derived contaminants/pollutants and therefore under a high selective pressure.

### Laboratory acclimation

*Ciona intestinalis* were collected by divers on 1–2 June 2016. The sea temperature was 17±1°C and upon collection, specimens were immediately transported under immersion and temperature-controlled conditions (17±1°C), to the aquatic facilities of the Sven Lovén Centre for Marine Infrastructure (Kristineberg, University of Gothenburg, Sweden). Upon arrival, specimens were taxonomically identified (i.e. at a species level) by diagnosing morphological traits under stereomicroscope observations (Brunetti et al., 2015). Subsequently, each sampled population was individually placed in rectangular shaped tanks (30 l total volume each, ∼1 adult specimen l^−1^) and flow-through supplied and fed with unfiltered natural seawater (NSW, flow rate ∼1 litre min^−1^). Specimens were laboratory acclimated for 15 days, under prevailing natural conditions [i.e. seawater temperature (16.93±0.61°C), salinity (26.54±2.18)]. Overhead artificial illumination was provided on a 12 h:12 h light:dark photoperiod, during the entire experimental period (Lambert and Brandt, 1967). Abiotic NSW parameters were monitored constantly (https://www.weather.mi.gu.se/kristineberg/en/data.shtml).

### Strip spawning and *in vitro* fertilization

**Fig. 2.**
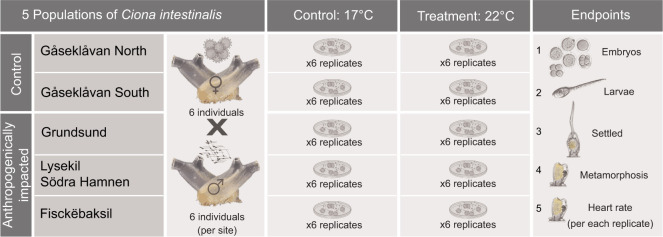
**Experimental design showing the cross design of gametes obtained from wild adult *C. intestinalis* collected at each studied location.** Twelve adults (6 males and 6 females) were collected from each of 5 locations. Fertilised eggs from each crossing per each site, were divided into 6 replicates per each temperature condition (control: 17°C and treatment: 22°C) and raised to juveniles. Five endpoints were scored from embryos to juvenile.

In order to collect gametes directly from adult individuals, strip spawning of *C. intestinalis* was performed, under stereomicroscope observation (Leica M2 16 A, Germany). For each population (*n*=5), a total of 12 adult individuals were dissected (Fig. 2). To avoid autologous contamination, oocytes were collected from 6 individuals used as functional females, and sperm from 6 functional males. Gametes were collected one at a time and separately from each individual using clean glassware apparatus. Oocytes were collected into Petri dishes (9 cm diameter, 1.3 cm high), filled with filtered (0.20 µm) and autoclaved NSW. Sperm was ‘dry’ collected (i.e. avoiding contact with seawater to prevent cell activation) into Eppendorf vials and kept on ice (−4°C). Afterwards, sperm was diluted (1:1000) and *in vitro* fertilization performed adding 10 µl ml^−1^ of oocyte suspension (Lambert, 2014). The morphology of the eggs was checked after 10 min, when the egg's shape changed transiently from spherical to elongated owing to second polar body formation. The resulting mixture was washed after ∼15 min with filtered and autoclaved NSW, in order to remove excess sperm. A detailed description of gametes collection and *in vitro* fertilization is described elsewhere (Lambert, 2014).

### Experimental exposure

After *in vitro* fertilization (∼15 min), offspring from the same cross was divided into 6 replicates across the two temperature treatments/conditions (Fig. 2), comprising 60 in total (i.e. 5 populations under 2 experimental temperature conditions). Each replicate consisted of a Petri dish [filled with 0.20 µm filtered, temperature pre-conditioned and autoclaved NSW (∼8 ml)] containing ∼300 fertilised eggs each, that were immediately allocated under experimental temperature conditions. Experimental exposure was performed under a controlled temperature environment (EVO Incubators, Friocell, USA) and consisted of control (17°C) and an increased temperature (22°C) condition mimicking a heatwave event, following Landis et al. (2012). This is equivalent to the category II extreme heatwave recorded in the sampling location in the summer of 2018 (please see discussion below). The partial pressure of O_2_ tension was measured in the filling jug, before the water was changed, to ensure adequate oxygenation was maintained within the Petri dishes (E5047, Radiometer, Copenhagen, Denmark).

### Early life stage development

Within each replicate, the number of fertilized eggs was recorded at 2 h post fertilization, against which the performance of the remaining analysed endpoints was scored. During the incubation period, Petri dishes were gently and frequently shaken, in order to minimize the risk of water stratification. To maintain water quality during *C. intestinalis* early life stage development, approximately two-thirds of the treatment water was changed every 24 h (NSW abiotic parameters were monitored throughout to ensure consistency). For each population, experimental condition and replicate, *C. intestinalis* development was verified and followed according to Hotta et al. (2007) (Fig. 2). Number of normally developed embryos was assessed after 5 h post fertilization (embryo development), as a proxy for population resilience towards experimental conditions (i.e. temperature, Kawaguchi et al., 2015). Number of actively swimming larvae (i.e. competent) was assessed at 18 h post fertilization (larvae development). At 24 h post fertilization (settlement success), the number of settled larvae (i.e. attached by the papillae to the substrate) was recorded (Chiba et al., 2004). Settled larvae that went through metamorphosis were checked after 60 h postfertilization (metamorphosis success), considering only those where heartbeat was evident (Chiba et al., 2004). Finally, heart rate of 4 viable post settled juveniles (per replicate) was assessed (juvenile heart rate), within 3 out of 6 randomly selected replicates (per population and experimental condition). All endpoints (exception for heart rate) were defined as ratios and calculated against the number of initial fertilized eggs. Heart rate was defined as the number of beats min^−1^; a single peristaltic wave (undulation) was considered one heartbeat and recorded at 30 s intervals. The pumping action of the ascidian heart is produced by a peristaltic wave and the direction of peristalsis reverses periodically (Chiba et al., 2004). All observations were performed under a binocular stereo microscope (model M2 16 A, Leica, Germany).

### Heatwave analysis

Marine heatwave occurrence and frequency were evaluated using a 30 year dataset [https://coastwatch.pfeg.noaa.gov/erddap/griddap/NOAA_DHW.html?CRW_SST%5B(2020-12-21T12:00:00Z)%5D%5B(58.375):(58.225)%5D%5B(11.575):(11.625)%5D&.draw=surface&.vars=longitude%7Clatitude%7CCRW_SST&.colorBar=%7C%7C%7C%7C%7C&.bgColor=0xffccccff; December 1990 to December 2020; US National Oceanic and Atmospheric Administration (NOAA) 2020; accessed 22 December 2020] for seawater surface temperature the study area. The data set was acquired from NOAA (daily optimum interpolation SST, v.2), at 5 km resolution produced daily in near real-time. The R package heatwaveR (https://cran.r-project.org/web/packages/heatwaveR) was used to detect and categorize marine heatwaves in the region applying the marine heatwave definition by Hobday et al. (2018).

### Statistical analysis

In order to assess if the overall response of each population significantly differed with treatments, we first performed a multivariate analysis of variance (MANOVA), using all quantified endpoints. Subsequently, each endpoint was modelled as a function of interaction between site conditions (anthropogenically impacted versus control) and temperature. Site condition was nested in the population level and subsequently modelled as random nested effects allowing for different intercepts at each site condition level (anthropogenically impacted versus control), within populations. We fitted generalised linear mixed effects models to test our hypothesis, using the nlme package (https://cran.r-project.org/web/packages/nlme), in the R environment (https://www.r-project.org/). The lme function allowed the modelling of heteroscedasticity of the within-error group via the ‘weights’ argument (Pinheiro and Bates, 2000; Zuur et al., 2009), accounting also for the lack of independence among endpoints for each sample (i.e. Petri dish) (Field et al., 2012). By applying this variance function structure, we allowed the model to adjust for standard errors and fit the heteroscedastic variance of the different populations. The success rate of each endpoint was scored against the initial number of fertilised eggs (obviously cleaved). Selection from saturated to reduced model was ranked according to Akaike information criteria and anova.lme function (https://cran.r-project.org/web/packages/nlme). Figures were created using ggplot2 package (https://cran.r-project.org/web/packages/ggplot).

## RESULTS

**Fig. 3.**
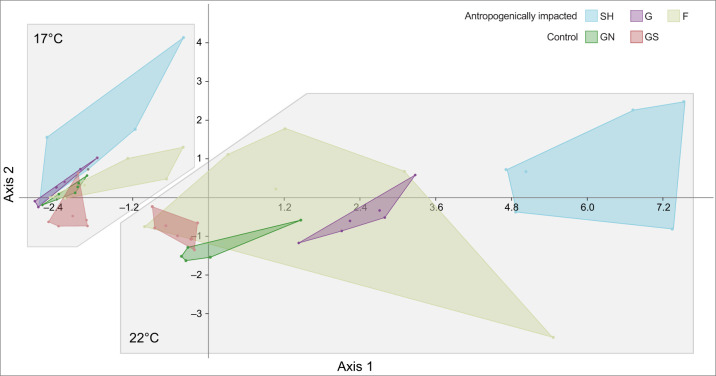
**MANOVA/CVA ordination plot showing the overall response of the analysed endpoints for each site and temperature condition (clusters).** Sampled populations: SH, Södra Hamnen; G, Grundsund; F, Fiskebackskil; GN, Gåseklåvan North; GS, Gåseklåvan South. Exposure temperature conditions: 17°C (control); 22°C (marine heatwave).

Temperature had a significant overall effect over the analysed endpoints, between sampled populations (MANOVA, λ Wilks=0.04; *P*=0.0003, Fig. 3). The first axis accounted for 88.99% of the observed variation, while the second axis just explained 7.36%. Under the controlled temperature condition, the overall response within anthropogenically impacted populations was less variable, in comparison to results obtained in the increased temperature group (Fig. 3). Control populations showed less variation, under both temperature conditions (Fig. 3).

The individual analysis of each endpoint (by mixed-effect models) showed that the interaction between site (anthropogenically impacted versus control) and experimental temperature conditions (17°C versus 22°C), was statistically significant for three of the analysed endpoints (Table 1): embryo development (*t*=5.399; *P*≤0.001), larva development (*t*=−5.398; *P*≤0.001) and metamorphosis success (*t*=−4.574; *P*≤0.001).

**Table 1.**
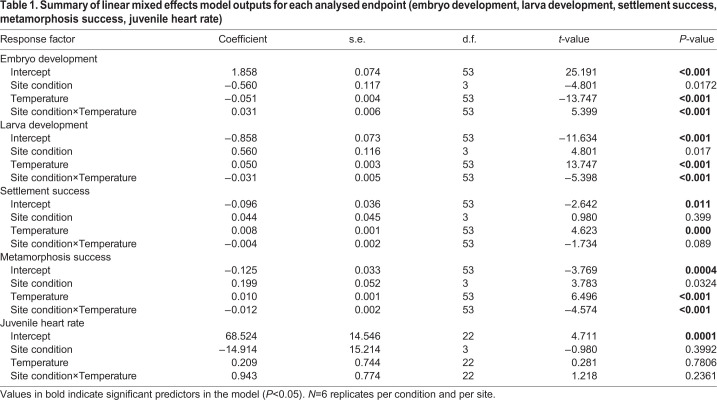
**Summary of linear mixed effects model outputs for each analysed endpoint (embryo development, larva development, settlement** **success, metamorphosis success, juvenile heart rate)**

**Fig. 4.**
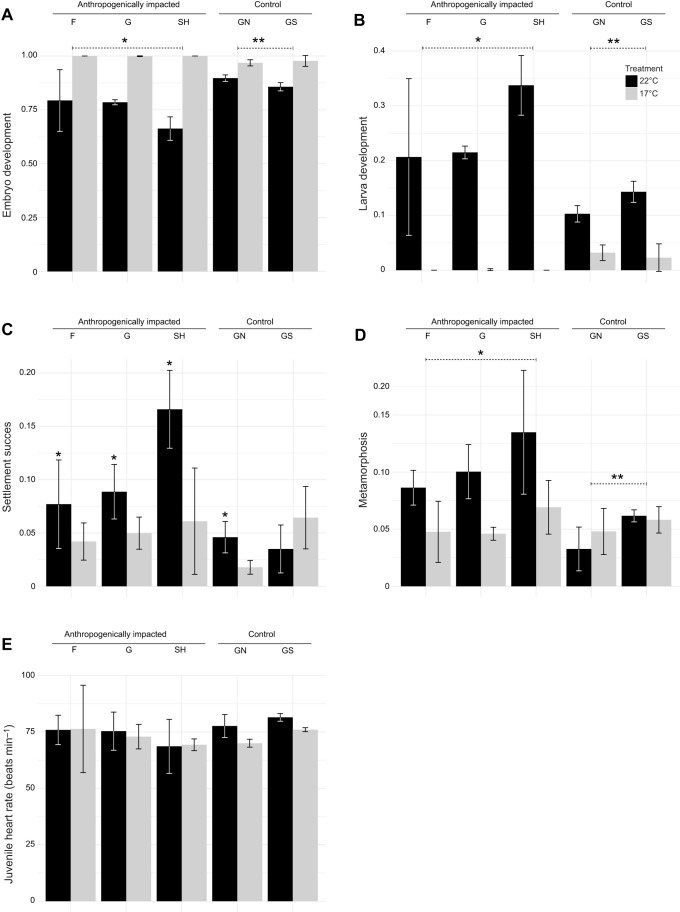
**Development performance of *C. intestinalis* early life stages, derived from populations of anthropogenically impacted and control sites, subjected to control (17°C) and simulated heatwave (22°C) temperatures.** Mean±s.e.m. are shown for each endpoint: (A) Embryo development; (B) larva development; (C) settlement success; (D) metamorphosis; (E) juvenile heart rate. Statistically significant differences from mixed effect models are indicated with asterisks when they are due only to the temperature condition; the asterisk accompanied by a dotted line, indicates observed differences due to the interaction of the two analyzed conditions (site and temperature). With the exception for juvenile heart rate, remaining analysed endpoints are expressed as ratios, scored against the initial number of fertilised eggs. SH, Södra Hamnen; G, Grundsund; F, Fiskebackskil; GN, Gåseklåvan North; GS, Gåseklåvan South.

Embryo development varied significantly among temperature conditions, with response being site dependent (Table 1). The interaction between site and temperature conditions was statistically significant (*t*=5.399; *P*≤0.011, Table 1). Anthropogenically impacted populations presented a lower ratio of normally developed embryos at 22°C, in comparison to control populations (Fig. 4A). Similarly, larva development response to temperature was dependent on site condition (*t*=−5.398; *P*≤0.001, Table 1). Although developed larvae increased at 22°C in all analysed populations, for anthropogenically impacted populations the ratio doubled in comparison to control populations (Fig. 4B). In addition, development of larvae from anthropogenically impacted populations was arrested under control temperature conditions (Fig. 4B).

Settlement success was significantly affected by temperature (*t*=4.623; *P*=0.0003, Table 1). Although site condition did not show a significant effect, a higher number of settled individuals was observed for anthropogenically impacted sites at 22°C (Fig. 4C). In addition, temperature did not have a significant effect on settlement success for Gåseklåvan South (a control site) (Fig. 4C). Site and temperature conditions had a significant combined effect (*t*=−4.574; *P*≤0.001, Table 1) on metamorphosis success of populations derived from anthropogenically impacted sites, showing a higher ratio at 22°C (Fig. 4D). In contrast, temperature condition did not affect metamorphosis success of *C*. *intestinalis* populations, derived from control sites (Fig. 4D). Heart rates did not show a significant overall response to the effect of temperature (*t*=−0.28; *P*=0.78, Table 1) and site condition factors (*t*=1.37; *P*=0.26, Table 1).

## DISCUSSION

Our results support the hypothesis that local habitat history of *C. intestinalis* populations can confer an advantage towards larval development during a marine heatwave event, assuming a selective pressure upon parental populations. We found a higher variability in development at early life stages in populations from anthropogenically impacted sites, which could offer an advantage for *C. intestinalis* as this implies a greater potential to adapt to environmental stressors (Beaman et al., 2016).

The high viability of embryos observed in our study is consistent with normal embryogenesis progression (Bellas et al., 2003; Hotta et al., 2007; Aguirre et al., 2014; Malfant et al., 2017). This empirically based result provides strong evidence that the observed effects in the later stages of development are due to the effects of the experimental treatments (site and temperature) and not to other factors related to gamete viability or the fertilization process. However, our data show that the development of embryos, larvae and metamorphosis success are the main early life stages whose response to thermal stress would be conditioned by the environmental history of the parental population, and is supported by the mixed model. Embryo development observed from control sites, outperformed anthropogenically impacted sites under thermal stress and additionally showed a more consistent overall response in comparison to anthropogenically impacted populations. This would be expected since embryonic development of *C. intestinalis* typically occurs, between 18°C and 23°C (Bellas et al., 2003; Rius et al., 2014). However, it is not uncommon that the exposure of a single stressor leads to immediate effects as we observed in fertilization after heat treatment in control animals (Lange and Marshall, 2017). Furthermore, the reduced development of embryos from anthropogenically disturbed sites, would be indicative of an additive effect of parental experience and heat treatment (Lange and Marshall, 2017). An abrupt temperature increase in an undisturbed population could interfere with *C. intestinalis* homeostasis and subsequent later stages of development as we observed in our study and coinciding also with previous reports (Dybern, 1965; Bellas et al., 2003; Kawaguchi et al., 2015; Malfant et al., 2017).

Larval development showed a positive response to the simulated heatwave but was highly conditioned by the environmental background of the population. Under control temperature conditions (17°C), the rate of competent larvae (that hatched and were actively swimming) was lower for anthropogenically impacted populations in comparison to control ones. Despite the higher performance of embryos in control populations, larvae development did not equally perform well. Our results coincide with other studies addressing thermal-, salinity- and pollution-induced stress in two invasive ascidians (*Styela plicata* and *Microcosmus squamiger*), that found fertilization and larval development to be highly sensitive, which in turn subsequently affected the early development of these species (Pineda et al., 2012). Additionally, the findings of Pineda et al. (2012) support our assumption that environmental exposure conditions of parental populations (anthropogenic pressure) most likely augmented and subsequently shaped their offspring's physiological response to induced stress, which supports previous observations related to the osmotic tolerance response (Renborg et al., 2014). However, our results suggest that the combined effect of environmental background and temperature could affect larval development, delaying the time from fertilization to hatching and thus affecting the progression of embryogenesis under controlled temperature conditions. Time from fertilization to hatching of *C. intestinalis* wild populations can take up to 18 h at 20°C, while at 16°C it could last 22 h (Kroiher et al., 1992; Matsunobu and Sasakura, 2015), which is congruent with our results regarding the control populations at the control temperature (17°C). Genetic association among fitness components of *C. intestinalis* early life stage demonstrated that offspring derived from ascidians with higher levels of larval viability had increased mortality during embryogenesis (Aguirre et al., 2014), which further supports our hypothesis, given the lower ratio of embryos that we observed.

In our study, larval settlement was examined 24 h post fertilization, i.e. 4 h post-hatching. Matsunobu and Sasakura (2015) estimated that settlement would start at approximately 3 h after hatching, regardless of temperature. We observed that larvae derived from anthropogenically impacted populations hatched within a short time period and settled, which explains why we observed settled larvae from anthropogenically influenced populations, in spite of the lower ratio of developed larvae. Some authors suggest a correlation between the induction of *C. intestinalis* metamorphosis (settlement and tail regression) and heat shock protein synthesis, after heat treatment above 20°C (Kroiher et al., 1992). Considering the heat treatment applied in our study (22°C), settlement could have been promoted by heat shock with different impact among populations: anthropogenically impacted sites had the same pattern of response but the same was not observed among control sites. Although mixed model analysis failed to find a site effect (anthropogenically impacted versus control), a site-specific variability among sampled control populations should not be disregarded. Furthermore, metamorphosis varied significantly with the interaction of temperature and the parental origin (anthropogenically impacted versus control). Although previous studies on ascidians have demonstrated that metamorphosis is not affected by temperature (Thiyagarajan and Qian, 2003; Matsunobu and Sasakura, 2015), our data show that the observed higher metamorphosis ratio (populations from anthropogenically impacted sites) and the interaction term of mixed effect models could be indicative of co-occurrence of extrinsic (e.g. temperature) and intrinsic (e.g. parental experience) factors which may have a significant overall effect on *C. intestinalis* metamorphosis (Svane and Young, 1989). Although for the majority of the analysed endpoints, *C. intestinalis* was revealed to be more responsive to heatwave treatment conditions, heart rate analysis did not provide evidence to explain the observed results related to either a population component or increased temperature. Moreover, the available literature suggests that heart rate of several ascidian species is mainly affected by osmotic stress (i.e. reduction in salinity), with a stronger effect being observed when combined with higher temperatures (Shumway, 1978; Dijkstra et al., 2008; Pineda et al., 2012; Malfant et al., 2017).

Ascidians (Tunicata, Ascidiacea) are widely distributed around the globe (e.g. Lambert, 2007; Shenkar and Swalla, 2011; Simkanin et al., 2016), and are able to adapt to specific local conditions. Our findings are particularly pertinent in two ways. Firstly, a sudden temperature increase can have an unpredicted outcome on invasion potential/dynamics of *C. intestinalis.* Current evidence indicates that non-native species perform better than native under extreme conditions (Stachowicz et al., 2002; Smale et al., 2011; Sorte et al., 2013; Rius et al., 2014; Gewing et al., 2019; Kenworthy et al., 2018). The greater tolerance to heat stress displayed by anthropogenically disturbed compared with control populations, shows that spreading of species reared in stressful habitat conditions could aggravate competitive asymmetries between species under extreme conditions such as heatwaves and climate change. Under harsh conditions ascidians are thought to not successfully complete early life stages of development because larvae may be capable of detecting the prevailing environment, withholding attachment to substrates in unfavourable conditions, and therefore delaying recruitment until more tolerable conditions (Bellas et al., 2004; Pineda et al., 2012). However, larva development, metamorphosis and settlement are enhanced under high temperatures (Gewing et al., 2019). Our study indicates that settlement and recruitment are significantly enhanced after being subjected to stressful conditions. This could be linked to the low levels of embryo development under heat treatment, suggesting that the stressor has an additive effect on anthropogenically impacted populations, selecting against the least viable individuals. By contrast, in populations that were not under environmental stress, the embryos would experience an apparent benefit with later detrimental consequences (Dybern, 1965; Bellas et al., 2003; Kawaguchi et al., 2015; Malfant et al., 2017). Secondly, the fact that such responses are observed in native populations, strengthens our conclusion regarding the influence of parental experience and the selective anthropogenically derived pressure of contamination/pollution over invasive potential success. Consequently, after overcoming such critical environmental conditions, resulting offspring are likely to shape variations or even differences in population-specific physiological traits (Magozzi and Calosi, 2015) and subsequently confer tolerance to different environmental conditions (Vercaemer et al., 2011; Renborg et al., 2014).

In our study, heat stress consistently induced a stronger development response in *C. intestinalis* derived from anthropogenically impacted populations in comparison to control populations. Such repeatable and directional change that favoured early life stages performance as a response to a specific environmental signal (i.e. temperature) could be, to some extent, indicative of adaptive phenotypic plasticity (Travis, 1994; Doughty and Reznick, 2004). In this sense, it has been observed that during *C. intestinalis* larval metamorphosis, a plastic response towards osmotic tolerance is driven by parental experience (Renborg et al., 2014). Conversely, the increased variability observed in *C. intestinalis* early life stages from control sites towards heat shock exposure, could suggest a lower plasticity and subsequently inherent ability to adapt to the imposed environmental stress. As such, this further supports our hypothesis that native species occurring in anthropogenically impacted sites are likely to become more successful invaders in comparison to those inhabiting sites which are not under anthropogenic influence. To the best of our knowledge, only one report indicates that temperature alone influences the developmental performance of juvenile *C. intestinalis* (Malfant et al., 2017).

Other environmental or unrelated factors that may be influencing early life stage development need to be considered as contributing components. Connectivity among *C. intestinalis* populations can modulate the flux of adaptive genetic variation from numerous areas and habitats (Ghabooli et al., 2013). The ability of *C. intestinalis* to disperse is crucially dependent on the larval competence and capability to settle (Rius et al., 2014). However, at the local scale, short-distance dispersal occurring by means of drifting *C. intestinalis* eggs, egg-strings and swimming larvae (Svane and Havenhand, 1993) limit the dispersion of this species, once the eggs and developing larvae are maintained in mucus strings (Petersen and Svane, 1995) and local oceanographic patterns constrain *C. intestinalis* populations connectivity (Johannesson et al., 2018). The expansion of global trade and maritime traffic will be a main contributor to the increasing rate of introduction of marine species worldwide (Shenkar and Rosen, 2018). Anthropogenically impacted sites, such as harbours and marinas, are some of the most vulnerable environments as they are directly exposed to the introduction of non-native species (Lambert and Lambert, 1998; Airoldi et al., 2015). Additionally, they can act as donor environments (Keller et al., 2011), creating pivotal breeding grounds for non-native species and subsequently sustain the biological spreading of non-native species to new areas (Simkanin et al., 2012; López-Legentil et al., 2015). These sites concentrate propagules of multiple origins and therefore increase the invasiveness potential of non-native species by introducing adaptive genetic variation from numerous areas and habitats (Ghabooli et al., 2013). However, these same anthropogenically impacted sites are subjected to high levels of anthropogenic disturbance (e.g. pollutants), which constitute well known stressors in the marine environment and powerful agents of selection, exerting high pressure on maximum physiological responses of organisms (Piola and Johnston, 2007; Levinton et al., 2003; Galletly et al., 2007). The occurrence of site-specific environmental conditions can expose populations to intense multiple generation selection (Medina et al., 2007), resulting in the evolution of subtle variations or even differences in physiological traits (Magozzi and Calosi, 2015) and subsequently giving rise to population-specific intransigence (Klerks and Weis, 1987). In fact, populations from different Scandinavian sites can exhibit different ranges of salinity tolerance for the development of fertilized eggs and larvae (Dybern, 1967).

**Fig. 5.**
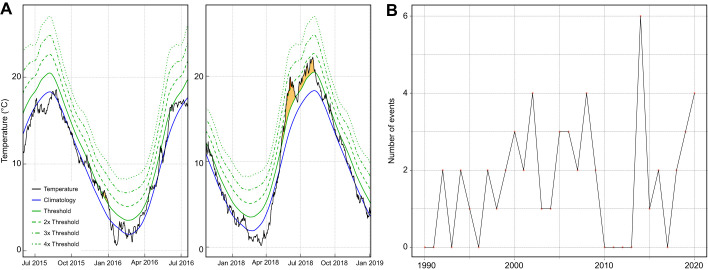
**Marine heatwave detection from a dataset of sea surface temperatures.** (A) Summer of 2018 category II marine heatwave. Marine heatwave detection in 2015–2016 (left) and 2018–2019 (right). Temperatures above threshold (90th percentile in relation to the long-term climatology) represented as yellow (category I) and orange (threshold 2×; category II) (Hobday et al., 2018). (B) Frequency of marine heatwave events within the study area. Marine heatwave characterization was performed using a 30-year dataset (https://coastwatch.pfeg.noaa.gov/erddap/griddap/NOAA_DHW.html?CRW_SST%5B(2020-12-21T12:00:00Z)%5D%5B(58.375):(58.225)%5D%5B(11.575):(11.625)%5D&.draw=surface&.vars=longitude%7Clatitude%7CCRW_SST&.colorBar=%7C%7C%7C%7C%7C&.bgColor=0xffccccff; December 1990 to December 2020, NOAA, 2020) for seawater surface temperature for the sampling location.

In the near future, not only are global trade and maritime traffic predicted to increase, but so is the frequency and magnitude of extreme warming events (Mann et al., 2017). Using data available from NOAA , we determined that during the 12 months prior to the sampling, only two moderate marine heatwaves took place in the area: on 18–22 December 2015 and 11–14 April 2016 (Fig. 5A,B). Moreover, we were able to demonstrate a 5°C increase in surface sea temperature, corresponding to category II and category III heatwaves. In fact, in 2018, a category II summer heatwave led to sea water temperature rising to 22.2°C (Fig. 5A). Furthermore, compiling data from marine heatwave detection NOAA dataset of sea surface temperatures for the last 30 years indicates that there have been numerous heatwaves occurring annually (Fig. 5B).

The relevance of our proposed research question is therefore evident within marine invasion ecology, but also in a broader context of global environmental change. Not only the studied area but other places along Central Europe would be experiencing similar scenarios, affecting many species. The brown seaweed *Fucus vesiculosus* and the seagrass *Zostera marina* seem to be fairly tolerant to short-term marine heatwaves (up to 5°C above mean temperature) in the Baltic Sea (Saha et al., 2020). Similarly, in a mesocosm study on the heatwave effect over temperate macrobenthic community of the western Baltic Sea, Pansch et al. (2018) observed that half of the species did not respond to heatwaves, and concluded that these are tolerant to short-term heatwaves. Furthermore, and in line with our results, heatwaves in general favoured suspension feeders that showed enhanced reproduction, suggesting that the subsequent survival of the more robust offspring (i.e. selection) could enhance thermal robustness over time (Pansch et al., 2018). Early development in other aquatic species, however, has been demonstrated to be negatively affected. For example, Seuront et al. (2019) showed decreased thermal tolerance under recurrent heat stress conditions resulting in mass mortality in the blue mussel *Mytilus edulis* along the northern French coast. Also, diminished reproductive rates of male and female pipefish have been linked in a rise in Swedish coastal waters from 10 to 15°C (Ahnesjo, 1995). Thus, although the extent of heatwave threat is large since it can drive different species-level responses that translate to the community level (Pansch et al., 2018), when combined with enhanced thermal tolerance linked to contamination/pollution, it can aggravate competitive asymmetries between species, triggering important shifts in community structure with further consequences for ecosystem functioning.

The present investigation demonstrates that *C. intestinalis* not only inhabits highly disturbed environments (i.e. contaminated/polluted sites), but early life stages can thrive under the additive pressure of heat shock exposure. Pollution is known to affect embryogenesis and lead to carry-over effects over species settlement and metamorphosis related processes in marine invertebrates (e.g. bryozoan *Watersipora subtorquata*, Ng and Keough, 2003; bryozoan *Bugula neritina*, Lange and Marshall, 2017; sea squirt *C. intestinalis*, Bellas et al., 2004). These are critical stages in species with complex life cycles, but as not all exposed animals are equally affected, the environmental pressure can act by selecting stronger phenotypes, shaping more resistant populations in subsequent generations (Foo and Byrne, 2016; Vihtakari et al., 2016). This is further supported by studies highlighting that tolerance to pollution and environmental stress are key determinants of invasion success (e.g. Kolar and Lodge, 2001; Braby and Somero, 2006; Olden et al., 2006). Nevertheless, empirical evidence showing that invasive species successfully cope under unfavourable condition is limited and still not conclusive (Prenter et al., 2004; Lenz et al., 2011; Faria et al., 2010; Bielen et al., 2016; Kenworthy et al., 2018), probably due to biases linked to the comparison between native versus alien species that are taxonomically close. Therefore, an intra-specific comparison among native populations, as we proposed here, has the potential to provide a more valuable insight regarding invasiveness success and abiotic tolerance. Moreover, in light of the overall higher performance in anthropogenically impacted populations under heat stress conditions, our study provides unquestionable empirical evidence to sustain the hypothesis of species-specific pre-adaptation in organisms that originate from harsh and/or fluctuating environments and consequently, that anthropogenically shaped populations would likely be more resilient to predicted future heatwaves scenarios, in comparison to those not exposed and derived from control sites.
